# Depression, anxiety and stress among female patients of infertility; A case control study

**DOI:** 10.12669/pjms.326.10828

**Published:** 2016

**Authors:** Lamia Yusuf

**Affiliations:** Dr. Lamia Yusuf, Assistant Professor of Gynaecology/Obstetrics, Rashid Latif Medical College, Lahore, Pakistan

**Keywords:** Infertility, Depression, Anxiety, Stress, Mental Health

## Abstract

**Objectives:**

Infertility, in many ways, is a very distressing condition that can have its impact on social and marital life of a couple. Depression, anxiety and stress associated with infertility may affect treatment and outcomes for such couples. The purpose of this study was to find out prevalence of depression, anxiety and stress among females suffering from infertility.

**Methods:**

One hundred females suffering from infertility as study subjects and 100 females accompanying them as controls were randomly selected from infertility clinic at Arif Memorial Teaching Hospital, Lahore, Pakistan. Females with diagnosed mental health issues and those from couples having male factor infertility were not included. Validated Urdu version of Depression, anxiety, stress scale (DASS) was used for assessment of depression, anxiety and stress scores. Results from both groups were compared and independent sample *t*-test was used to analyze the results.

**Results:**

There was high prevalence of depression, anxiety and stress among females suffering from infertility compared to females in control group (*p* < 0.05). Level of education did not appear to have any positive effect on these scores. Similarly, results did not appear to change when occupations of infertile females were used for stratified analysis.

**Conclusion:**

Depression, anxiety and stress are very common among females suffering from infertility. Healthcare professionals should consider psychological counseling, and psychiatric help if required, when they offer fertility treatment for such females.

## INTRODUCTION

Having children is one of the most likely wishes any couple can have and being unable to bear one despite having regular and unprotected intercourse for a year, is defined as infertility. Prevalence of infertility varies all over the world, largely depending upon the cultural and familial values.^1^ It is estimated that about 10% of the couples suffer from infertility, due to one or the other reason and in many of these couples cause remains unidentified.^2^

It is not uncommon for an infertile couple to develop mental health problems. The estimated prevalence of mental health problems ranges from 30% to 80% as reported in different studies and is linked to the cause and duration of infertility and number of attempts at different treatment options.^3^-^5^ Psychological impact of infertility may range from inferiority complex and stress to interpersonal relationships to major depression and anxiety.^6^,^7^

Females are more likely to suffer from psychological disturbances, especially in societies where females are mostly accused to be the reason for couple’s inability to conceive and cultural and social pressures and norms are one of the most important contributing factors in the development of these psychological issues. In addition, education and employment status of the female partner are among the influencing factors.^1^,^6^,^8^,^9^

In some societies e.g. Muslim societies, childlessness can especially be very distressing for infertile females because their religion and culture allow men to have more than one wives at the same time and female’s inability to conceive gives them a pretty good excuse to remarry.^10^

Pakistan has a culture where having children is considered of utmost importance for any couple. Expectations from society, family and friends put the couple, especially the female, in a very awkward situation. Men are often pressurized to for a second or multiple marriage and this adds to the psychological problem for the females.

Prevalence of psychological ailments among infertile Pakistani females has not been studied so far. So, this study was designed to assess the magnitude of depression, anxiety and stress among infertile females.

## METHODS

This case control study was conducted at Arif Memorial Teaching Hospital Lahore from February 2015 and August 2015. After approval from the hospital’s Ethics Committee, 100 female patients, with diagnosed female factor infertility, attending infertility clinic were included in the study and 100 fertile females who were accompanying the infertile patients were recruited in the study as controls. Females from couples who had male factor infertility and those with diagnosed mental health issues were not included in the study. A written and informed consent was obtained from all the patients after explaining the purpose and method of the study.

Socio-demographic information including age, occupation, educational level and presence or absence of pressure from family were obtained from the respondents. Depression, Anxiety Stress Scale (DASS) was used to collect data regarding psychological impact of infertility.

### Depression Anxiety Stress Scales (DASS)

To measure negative emotional states of depression, anxiety and stress, a validated Urdu version of 42-item DASS was used. Scores on each item can range from 0, indicating no symptomatology, to 3, indicating a severe level of symptomatology. Total score for each of the negative emotional states were calculated separately.^11^ DASS scoring was classified using the following classification.

**Table:** 

	Depression	Anxiety	Stress
Normal	0-9	0-7	0-14
Mild	10-13	8-9	15-18
Moderate	14-20	10-14	19-25
Severe	21-27	15-19	26-33
Extremely Severe	28+	20+	34+

### Statistical Analysis

Categorical variables i.e. age group, level of education and occupation were analyzed using chi-square test and are presented as frequencies and percentages. Depression, Anxiety and Stress Scores were analyzed using independent sample *t-*test and are presented as mean and standard deviation (SD). Stratified analysis was done to rule out effect of confounders.

## RESULTS

### Sociodemographic Characteristics

Most of the subjects included in the study were between 20-30 years of age (63.5%) and 31-40 years being the second most common age group (31.5%). The frequency of educated subjects was high in both control and study groups with 39% and 23% having 10 or less years of education and 23% and 40% having 12-14 years of education. The number of uneducated participants was 38 and 19 in control and study groups respectively. Only 18 respondents out of 200 had 16 years or higher education (n = 200). Percentage of house wives was higher (71% and 80%), employed being second in number (21% and 18%) and self-employed being the least common (8% and 2%).

**Fig.1:**
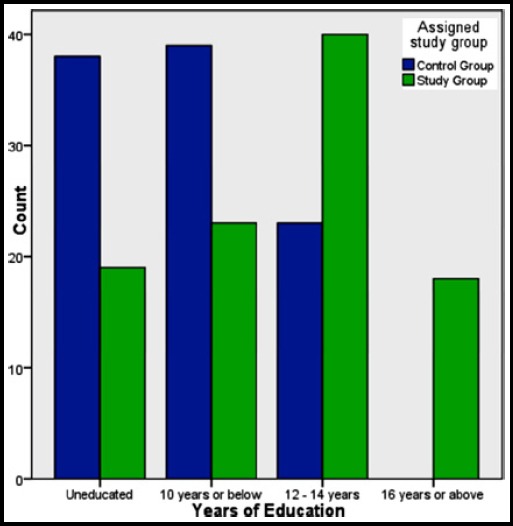
Level of education in two groups.

**Table-I:** Age group distribution in two groups

Assigned Study Group			

Characteristic		Control Group (n)	Study Group (n)
Age Group	20-30	63	64
	31-40	28	35
	> 40	9	1
Years of Education	Uneducated	38	19
	10 years or below	39	23
	12-14 years	23	40
	16 years or above	0	18
Occupation	House wife	71	80
	Employed	21	18
	Self Employed	8	2

### Depression Score

Results show that 79% of the patients with infertility had some degree of depression and 49% of the study group subjects had moderate to severe degree of depression and 10% had extremely severe depression. This was higher compared to control group where only 9% had mild depression and no subjects were found having higher degrees of depression. Mean Depression score in control group was 3.90 (SD ±4.165) in contrast to study group score16.14 (SD ±8.304) (*p* = 0.00). Stratified analysis considering years of education and occupation showed that results were not different in both groups, with higher depression scores in study group for all levels of education and occupation subgroups (*p* < 0.05).

### Anxiety Score

Mean Anxiety scores in Study group was higher (14.63 SD ±8.085) compared to study group (3.69 SD ±3.240) (*p* = 0.00). 41% subjects in study group had moderate to severe anxiety and 29% had extremely severe anxiety, whereas in control group 15% had mild and only 1% had moderate anxiety and no subject had severe or extremely severe anxiety. Similar findings were observed after stratified analysis for all occupation and level of education subgroups, with higher anxiety scores in study group (*p* < 0.05).

### Stress Score

Stress scores were higher in the study group (Mean stress score 19.72 ± 9.192) compared to control group (Mean stress score 5.87 ± 4.952) (*p* = 0.00). Only 5% subject in control group had mild degree of stress and none had higher degree of stress. In study group, 69% subject had some degree of stress (mild stress in 14%, moderate stress in 21%, severe stress in 29% and extremely severe stress in 5%). Stratified analysis revealed the same results for all level of education and occupation subgroups where higher stress scores were found in study group individuals compared to study group (*p <* 0.05).

## DISCUSSION

This study was conducted to find the magnitude of depression, anxiety and stress among females suffering from infertility, at a tertiary care hospital, in suburbs of Lahore Pakistan.

Infertility, for a Pakistani couple, is considered to be a havoc due to cultural and familial issues. Having children is every couple’s dream and for some families, this is of highest importance. For a married female, being childless can be disastrous especially because this gives males a reason to go for second (or more) marriage which is not very uncommon in this society and is a nightmare for a female. This socio-cultural pressure may lead to development of psychiatric symptoms in childless females.

Alhassan A et al. reported 62% infertile females having depression^1^ in Ghana. Similarly, Guerra D et al. found prevalence of depression to be 69% among infertile women^12^ in China. The prevalence of depression has been found to be 79% in our study, which is a bit higher compared to the two above mentioned studies. This higher prevalence of depression may have been contributed by the socio-cultural and religious norms that allow husbands for multiple marriages and the wife being infertile provides them with a reason for this decision. In addition, having a child, that too a male child, gives the females a sense of pride and a security for their old age. Higher rates of depression among infertile females have been reported from Japan^2^ and Gambia^13^ as well.

**Table-II:** Comparison of depression, anxiety and stress scores.

	Assigned study group	Mean	Std. Deviation	p-value
Depression Score	Control Group	3.90	4.165	
	Study Group	16.14	8.304	0.00
Anxiety Score	Control Group	3.69	3.240	
	Study Group	14.63	8.085	0.00
Stress Score	Control Group	5.87	4.952	
	Study Group	19.72	9.192	0.00

Our study showed that 70% of infertile females had a varying degree of anxiety and 58% of these had moderate to severe anxiety. Lawson AK et al. and Allen HT and Kraaji V et al. have shown higher rates of anxiety in patients undergoing infertility treatment.^14^-^16^ Similarly, stress scores have been found to be higher in infertile women by Luk BH et al, Dooley M et al and El Kissi Y et al have shown higher prevalence of stress among infertile females, in their studies, which is consistent with our finding (69%).^3^,^4^,^17^

One interesting finding from our study was that among infertile females, depression, anxiety and stress scores were higher no matter what their level of education was (*p <* 0.05). This differs from the results from Alhassan A et al.,^1^ who showed higher rates of psychological symptoms among infertile women with no or very little formal education.

Our study was not a prospective study (which was a limitation of this study) and we did not compare the depression, anxiety and stress score with those of patients suffering from other chronic illnesses. So, due to these limitations, results of the current study may not be generalized.

## CONCLUSION

The results from current study suggest very high rates of depression anxiety and stress among patients suffering from infertility. Further prospective studies comparing psychological stress of infertile females with females suffering from other chronic diseases and those exploring other factors that may be responsible for stress symptoms are suggested to confirm these results.
